# Increased gastric chromogranin A cell density following changes to diets of patients with irritable bowel syndrome

**DOI:** 10.3892/mmr.2014.2498

**Published:** 2014-08-18

**Authors:** TAREK MAZZAWI, DORIS GUNDERSEN, TRYGVE HAUSKEN, MAGDY EL-SALHY

**Affiliations:** 1 Section for Gastroenterology, Department of Medicine, Stord Helse-Fonna Hospital, Stord, Norway; 2 Section for Gastroenterology, Institute of Medicine, Bergen University, Bergen, Hordaland, Norway; 3 Department of Research, Helse-Fonna, Haugesund, Rogaland, Norway

**Keywords:** diet, computerized image analysis, chromogranin A, guts endocrine cells, immunohistochemistry

## Abstract

The gut endocrine cells control and regulate several functions of the gastrointestinal tract. They have been reported to be abnormal in irritable bowel syndrome (IBS), with alterations occurring in several functions regulated by these cells. Furthermore, it has been established that gut endocrine cells interact with the gut lumen contents, particularly the nutrients. The present study was undertaken to establish whether the positive outcome of dietary guidance observed in patients suffering from IBS is associated with a change in gastric endocrine cells. A total of 46 patients with IBS participated in the present study, of which 14 completed all aspects. These patients included nine females and five males with a mean age of 34 years (range, 20–45 years). In the healthy control group, nine females and five males, with a mean age of 54 years (range 26–70 years) were selected. The patients and controls underwent gastroscopy with biopsy samples taken from the corpus and antrum of the stomach. Each patient attended three sessions that lasted ~45 min each, and received individual guidance on their dietary management. The patients followed the diet prescribed for a minimum of three months, then further samples were taken using a method similar to that used for the initial biopsies. The biopsy samples were immunostained using the avidin-biotin complex method for chromogranin A (CgA) and quantified by computerized image analysis. The patients with IBS presented a low density of CgA compared with the controls. The density of CgA increased in these patients following dietary guidance and changes in food intake. The present observations emphasized the interaction between food intake and gut endocrine cells. The current study also suggests that the positive effects of dietary guidance may be attributed to changes in gut endocrine cell density.

## Introduction

The gut endocrine cells constitute the largest endocrine organ in the body and consist of at least 15 types of endocrine cell (1–3). In addition to the endocrine mechanism of action, the biologically active substances secreted by these cells act locally on nearby structures through paracrine signaling (4,5). The endocrine cells are scattered among the epithelial cells that line the gut lumen. They have specialized microvilli that project into the lumen and function as sensors for the gut contents (mostly for nutrients), and they respond to luminal stimuli by releasing their hormones into the lamina propria (6–18).

The stomach comprises of four types of endocrine cells, which produce serotonin, somatostatin, ghrelin and gastrin (5). While serotonin and somatostatin-secreting cells are present in the corpus and the antrum of the stomach, ghrelin-secreting cells are localized to the corpus and gastrin-secreting G cells are located in the antrum (19).

Chromogranin A (CgA) is a common marker for gut endocrine cells (20–22). In a previous study, the density of CgA-secreting cells was observed to be abnormal in the stomach of patients with irritable bowel syndrome (IBS) (23). Further studies have demonstrated that the densities of all the gastric endocrine cells are affected in patients with IBS (24).

Dietary management guidance given to patients with IBS has been indicated to reduce symptoms and improve the quality of life (25,26). The endocrine cells of the gut are stimulated by the contents of the gut lumen, particularly nutrients (27,28). Therefore, the present study was undertaken to investigate whether the positive effects of dietary management guidance observed in patients with IBS are associated with changes in the densities of endocrine cells in the stomachs of these patients.

## Materials and methods

### Patients and controls

Patients that were referred to our clinic at Stord Helse-Fonna Hospital (Stord, Norway) and fulfilled the Rome-III criteria (29) for IBS diagnosis were considered for inclusion in the current study. Females and males aged between 18 and 70 years were selected. Pregnant or lactating females, and patients with organic gastrointestinal or other systemic diseases, a history of drug abuse, or serious psychiatric disturbances were excluded. Patients that had undergone previous abdominal surgery, with the exception of appendectomy, caesarean and hysterectomy, were also excluded.

The control group included healthy subjects that underwent gastroscopy due to the following reasons: Gastrointestinal bleeding, where the source of bleeding was identified as hemorrhoids (n=3) or angiodysplasia (n=1); and health concerns as a result of diagnosis of a family member with gastrointestinal cancer (n=10). The control group consisted of nine females and five males with a mean age of 54 years (range, 26–70 years).

The present study was performed in accordance with the year 2000 edition of the Declaration of Helsinki and approved by the Regional Committee for Medical Research Ethics of West Norway. All patients submitted oral and written consent.

### Study design

A total of 46 patients participated in the present study, including 35 females and 11 males with a mean age of 35 years (range, 18–69 years). The patients were subjected to physical examinations and blood tests in order to exclude inflammation, infection or other organic diseases. Additionally, these patients underwent colonoscopy with segmental biopsy samples to exclude microscopic colitis. Each patient was scheduled for three sessions of individual dietary management guidance with an experienced nurse, lasting ~45 min each. These sessions were conducted with intervals of at least 2 weeks. The patients underwent gastroscopy prior to the first session and 3–9 months (median 4 months) following the third session of dietary management guidance.

### Individual dietary management guidance

In the sessions, information was provided orally using charts, in addition to written illustrated information. The initial session included general information regarding IBS and the importance of regular and healthy eating habits. The diets that worsen IBS symptoms, such as insoluble dietary fibers and the poorly absorbed highly fermentable oligosaccharides, disaccharides, monosaccharaides and polyols (FODMAPs) were explained. The patients were encouraged to consume dairy products daily and were informed that milk and dairy products do not provoke IBS symptoms. The patients were required to write a diary, in which they recorded their daily food and drink intake, the frequency and degree of abdominal pain, abdominal distension, stool frequency and consistency, for 2 weeks. In addition the patients were asked to test a protein-, fat- or carbohydrate-rich/poor diet. In the second session, the information given in the first session was briefly repeated. The nurse and the patient discussed the symptom-triggering items based on the information noted in the patient’s diary. The patients were then advised to alter the proportions of protein, fat and carbohydrate, avoid items rich in FODMAPs and insoluble fibers, and consume vegetables and fruits containing less FODMAPs and insoluble fibers. During the last session the patients informed the nurse of their experience of dietary management. The nurse, along with the patient, designed a suitable diet, which was strictly followed by the patient until the end-point of the study.

### Gastroscopy, tissue sampling, histopathology and immunohistochemistry

Following an overnight fast, the patients and control subjects underwent standard gastroscopy. Four biopsy samples were taken from the corpus (major curvature) and another four biopsy samples from the antrum of the stomach. Furthermore, four biopsy samples were taken from the duodenum in order to exclude celiac disease.

The biopsy samples were fixed in 4% buffered paraformaldehyde overnight, embedded in paraffin wax, and were then cut into 5-μm sections. Biopsy samples from the stomach and duodenum underwent histopathological examinations. Biopsy samples from the corpus and antrum were stained with hematoxylin and eosin and immunostained with the avidin-biotin complex method using a Vectastain ABC kit (Vector laboratories, Burlingame, CA, USA) and the chromogen 3,3′-diaminobenzidine peroxidase substrate (DAB) kit (Vector Laboratories) as described previously (30). Briefly, the sections were incubated for 2 h at room temperature with a monoclonal mouse anti-N-terminal of purified CgA primary antibody (code no. M869; Dako, Glostrup, Denmark) diluted 1:1,000. Following incubation, the sections were washed in phosphate-buffered saline (PBS; pH 7.4) and incubated for 30 min at room temperature with biotinylated swine anti-mouse IgG diluted to 1:200 (Dako). The slides were washed with PBS, and incubated for 30 min with avidin-biotin-peroxidase complex diluted 1:100, and then submerged in 3,3′-diaminobenzidine, followed by counterstaining with hematoxylin.

### Computerized image analysis

The density of chromogranin A in the corpus and antrum of patients with IBS and controls was measured using Olympus Cell^D software, Olympus, (Tokyo, Japan). The number of chromogranin A positive cells and the area of the epithelial cells were measured in 10 randomly selected fields, magnification, ×40. At this magnification each field represents a tissue area of 0.14 mm^2^. The chromogranin A cell density was expressed as the number of cells/mm^2^ of the epithelium. All quantification was conducted by the same scientist (Dr Tarek Mazzawi), who was blinded to the identity of the sections.

### Statistical analysis

The paired t-test was used to compare the results of patients prior to and following dietary guidance with results in the control subjects. The data are presented as the mean ± standard error. P<0.05 was considered to indicate a statistically significant difference.

## Results

### Patients and controls

A total of 25 patients withdrew their consent at various stages of the study. The majority of these were due to a lack of motivation when their symptoms improved following dietary guidance and/or unwillingness to undergo a second gastroscopy. Two patients were excluded due to non-compliance. Five patients were excluded as they were diagnosed with celiac disease (n=2), lupus (n=1), became pregnant (n=1) or moved abroad (n=1) during the course of the study. Thus, fourteen patients completed the study. These patients consisted of nine females and five males with a mean age of 34 years (range, 20–45 years). Biopsies from one female patient were obtained only from the antrum.

### Gastroscopy, histopathology and immunohistochemistry

The macroscopic appearance of the esophagus, stomach and duodenum was normal in patients and controls. Histopathological examination displayed normal histology of the stomach and duodenum in patients and controls. CgA immunoreactive cells were identified in the mucosa of the stomach in the two groups. These cells were either basket- or flask-shaped, and a number of them had a long basal cytoplasmic process.

### Computerized image analyses

#### Corpus

**Figure 1:**
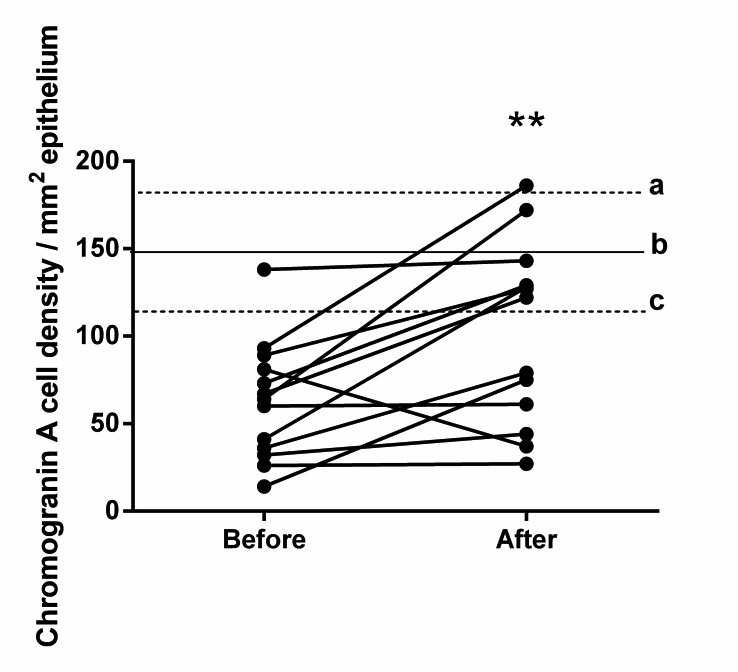
CgA cell density in the corpus of patients with IBS before and after receiving dietary management guidance. The dashed lines (a and c) represent the upper and lower limits of the 95% confidence interval of the mean in the control subjects. Line b represents the mean cell density of CgA in control subjects. ^**^P<0.01. CgA, chromogranin A; IBS, irritable bowel syndrome.

**Figure 2:**
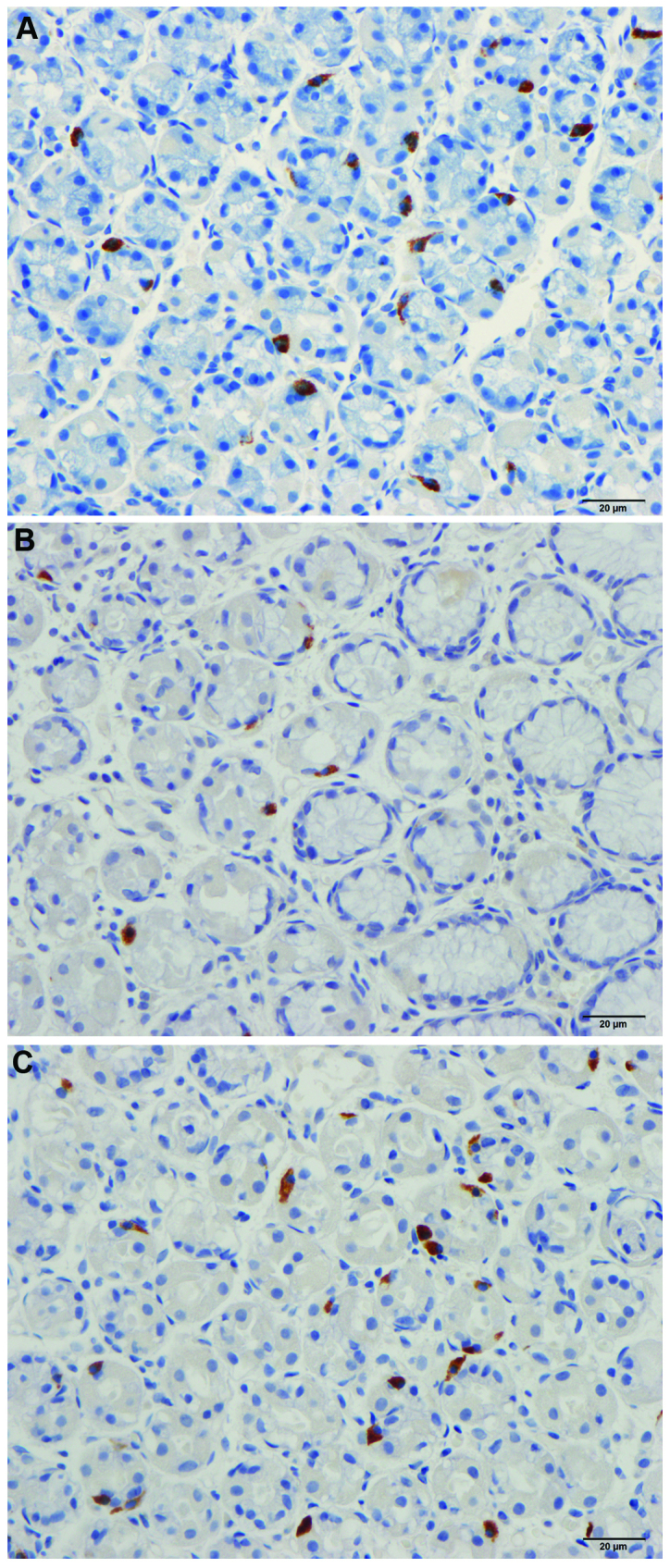
CgA immunoreactive cells in the corpus of (A) a control subject and an IBS patient (B) prior to and (C) following guidance on dietary management. The density of CgA cells prior to diet management is lower in IBS patients compared with controls, whereas the density in patients with IBS is almost the same as in controls following diet management. CgA, chromogranin A; IBS, irritable bowel syndrome.

The mean density of CgA-secreting cells in controls was 147.9 cells/mm^2^ (95% CI: 113.8–182). The densities of the CgA-secreting cells in patients with IBS prior to and following dietary guidance were 62.6±9.3 and 102.3±14.3 cells/mm^2^, respectively (Figs. 1 and 2). The paired t-test indicated a significant increase in the densities of CgA-secreting cells in IBS patients following dietary guidance (P=0.0064).

#### Antrum

**Figure 3:**
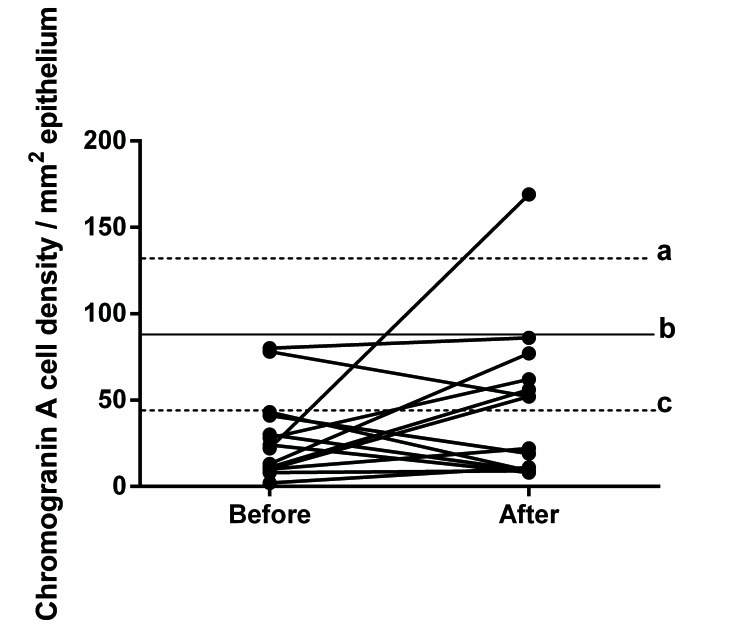
CgA cell density in the antrum of patients with IBS before and after receiving dietary management guidance. The dashed lines (a and c) represent the upper and lower limits of the 95% confidence interval of the mean in the control subjects. Line b represents the mean cell density of CgA in control subjects. CgA, chromogranin A; IBS, irritable bowel syndrome.

**Figure 4:**
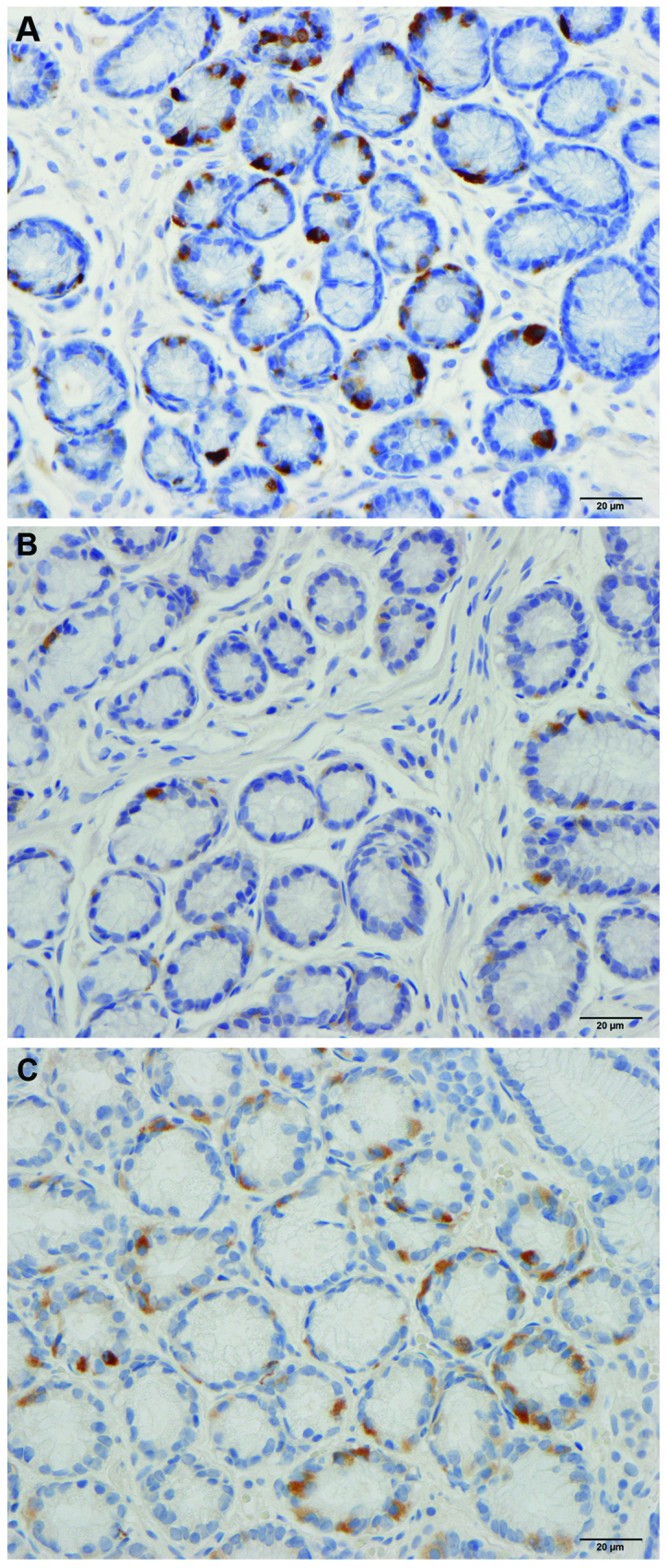
CgA immunoreactive cells in the antrum of (A) a control subject and an IBS patient (B) prior to and (C) following guidance on dietary management. The density of CgA cells prior to diet management is lower in IBS patients compared with controls, whereas the density in patients with IBS is almost the same as in controls following diet management. CgA, chromogranin A; IBS, irritable bowel syndrome.

The mean density of the CgA-secreting cells in the antrum was 87.7 cells/mm^2^ (95% CI: 43.8–131.7). The densities of the CgA-secreting cells in patients with IBS prior to and following dietary guidance was 28.5±6.5 and 46.5±11.1 cells/mm^2^, respectively (Figs. 3 and 4). No significant difference in the densities of CgA-secreting cells was detected in IBS patients prior to and following dietary guidance (P=0.2).

## Discussion

Patients with IBS are recognized to have a high dropout rate in clinical studies (26,31). The design of the present study involved invasive gastroscopy examinations, and adhering to a strict diet for a minimum of 3 months. This design may have contributed to the high rate of dropout in addition to the non-compliance experienced in the current study. Additionally, exclusions due to unexpected events occurring during the study, such as pregnancy, moving abroad or other diagnoses contributed to the low number of patients that fulfilled the criteria required to complete the study. Regardless of the small sample size, the present study indicated a clear effect on gastric endocrine cell density following changes in diet.

The density of CgA-secreting cells in the stomach (in the corpus and antrum) was abnormal in IBS patients in the present study prior to receiving dietary guidance, similar to results reported in a previous study (23). Following dietary guidance and a change of diet, the density of CgA-secreting cells significantly increased in the corpus towards the values observed in healthy controls. With regards to the antrum, these changes were also observed but did not reach a significant level. This increase demonstrates that changes in food intake via dietary guidance alters the density of the total gastric endocrine cells towards a normal level.

In a previous study on the same cohort of patients investigated in the present study (25), a similar dietary program resulted in a reduction of the symptoms and an improvement in the quality of life of the patients. The observation in the current study, that dietary changes can promote a density of endocrine cells that is more similar to that of healthy patients, suggests that these changes may be one of the causes for the amelioration of symptoms and consequently the improvement in the quality of life in the patients with IBS of the previous study. The findings of the present study support the hypothesis that gut endocrine cells are important in the pathogenesis of IBS (32,33).

Gut endocrine cells have microvilli extending to the lumen that act as sensors for the contents of the gut and respond to the luminal stimuli (32). It has been reported that each intestinal crypt contains 4–6 pluripotent cells that differentiate through a series of cellular precursors into all epithelial cell types, including the endocrine cells (34–43). This differentiation is rapid and takes 2–4 days (44,45).

In conclusion, the current study demonstrated that the change in diet with the consequent change in the contents of the gut lumen may be the cause of alterations to the differentiation of endocrine cells observed in patients with IBS, resulting in an increase in the density of endocrine cells.
